# Lung histopathologic clusters in severe COVID-19: a link between clinical picture and tissue damage

**DOI:** 10.1186/s13054-021-03846-5

**Published:** 2021-12-13

**Authors:** Maddalena Alessandra Wu, Gianluca Lopez, Manuela Nebuloni, Davide Ottolina, Jonathan Montomoli, Luca Carsana, Tommaso Fossali, Antonio Castelli, Roberto Rech, Chiara Cogliati, Emanuele Catena, Riccardo Colombo

**Affiliations:** 1 Division of Internal Medicine, ASST Fatebenefratelli Sacco, Milan, Italy; 2 School of Pathology, University of Milan, Milan, Italy; 3 Pathology Unit, ASST Fatebenefratelli Sacco, Milan, Italy; 4 Department of Biomedical and Clinical Sciences, University of Milan, Milan, Italy; 5 Division of Anesthesiology and Intensive Care, ASST Fatebenefratelli Sacco, Milan, Italy; 6 Division of Anesthesiology and Intensive Care, Ospedale Degli Infermi, Rimini, Italy

**Keywords:** COVID-19, Lung injury, Positive-pressure ventilation, Histology, Pneumonia

## Abstract

**Background:**

Autoptic pulmonary findings have been described in severe COVID-19 patients, but evidence regarding the correlation between clinical picture and lung histopathologic patterns is still weak.

**Methods:**

This was a retrospective cohort observational study conducted at the referral center for infectious diseases in northern Italy. Full lung autoptic findings and clinical data of patients who died from COVID-19 were analyzed. Lung histopathologic patterns were scored according to the extent of tissue damage. To consider coexisting histopathologic patterns, hierarchical clustering of histopathologic findings was applied.

**Results:**

Whole pulmonary examination was available in 75 out of 92 full autopsies. Forty-eight hospitalized patients (64%), 44 from ICU and four from the medical ward, had complete clinical data. The histopathologic patterns had a time-dependent distribution with considerable overlap among patterns. Duration of positive-pressure ventilation (*p* < 0.0001), mean positive end-expiratory pressure (PEEP) (*p* = 0.007), worst serum albumin (*p* = 0.017), interleukin 6 (*p* = 0.047), and kidney SOFA (*p* = 0.001) differed among histopathologic clusters. The amount of PEEP for long-lasting ventilatory treatment was associated with the cluster showing the largest areas of early and late proliferative diffuse alveolar damage. No pharmacologic interventions or comorbidities affected the lung histopathology.

**Conclusions:**

Our study draws a comprehensive link between the clinical and pulmonary histopathologic findings in a large cohort of COVID-19 patients. These results highlight that the positive end-expiratory pressures and the duration of the ventilatory treatment correlate with lung histopathologic patterns, providing new clues to the knowledge of the pathophysiology of severe SARS-CoV-2 pneumonia.

**Supplementary Information:**

The online version contains supplementary material available at 10.1186/s13054-021-03846-5.

## Introduction

SARS-CoV-2 infection causes a systemic disease, namely coronavirus 2019 disease (COVID-19), with multiple organ involvement [1–3]. However, the respiratory system is undoubtedly the main viral target, hence the name “severe acute respiratory syndrome,” and lung injury is the leading cause of death.

From the onset of the pandemic, some studies have described the postmortem findings, aiming at unveiling the characteristics and evolution of pulmonary alterations and their role in the pathogenesis of this complex condition. Autoptic findings usually reveal variable degrees of diffuse alveolar damage (DAD): Acute-phase DAD (during the first week after the pulmonary injury) usually shows exudative features with interstitial widening, formation of intra-alveolar edema, thickened alveolar septa, and perivascular lymphoplasmacytic infiltration, while in late-phase (one to several weeks) DAD proliferative features prevail with pneumocyte hyperplasia inducing interstitial thickening and collapsed alveoli, which may finally develop into organizing patterns (weeks to months), with marked fibroblastic proliferation and fibrosis, sometimes even leading to squamous metaplasia [4–8]. Even though the DAD pattern is not found exclusively in COVID-19, exudative or proliferative DAD development is a key pathophysiological mechanism in lung injury induced by SARS-CoV-2.

The characteristics of DAD evolve during the disease course. However, there may be high spatial and temporal heterogeneity, since features which can be usually found in the acute phase may coexist with features of the late phase and the relative extension of each pattern may be highly variable [4–8].

Besides damage of epithelial cells, SARS-CoV-2 has been proved to cause vascular alterations, with virions identified in the endothelial cells and hyperpermeability due to disruption of inter-endothelial junctions [4]. Endothelialitis, due to direct damage by SARS-CoV-2 and to multiple factors such as local/systemic inflammatory response and hypoxia, also leads to switching to a procoagulant phenotype of injured endothelial cells. Hence, it promotes the so-called immunothrombosis, leading to macrothrombosis (e.g., pulmonary embolism) and microthrombosis, often detected in autoptic samples [9–13]. These mechanisms are crucial in the immunopathogenesis of respiratory failure, which characterizes severe COVID-19.

Although autoptic pulmonary findings have already been described [8], there is still a significant dearth of knowledge on the correlation between clinical data and histopathologic pulmonary damage in patients who died from severe COVID-19.

This study aims to investigate the relationship between clinical–biochemical–radiological characteristics and histopathologic features in patients hospitalized for severe SARS-CoV-2 infection.

## Methods

This was a retrospective cohort observational study on consecutive patients who died from COVID-19 between February 29 and June 30, 2020, whose autopsies were conducted by the Pathology Unit at Luigi Sacco Hospital in Milan, the referral center for highly transmissible diseases in northern Italy.

### Patient samples

All patients had SARS-CoV-2 infection, confirmed by real-time PCR analysis on throat swab samples taken at the time of hospital admission or by real-time PCR performed on autoptic samples.

The inclusion criteria were death due to SARS-CoV-2 infection and full thoraco-abdominal autopsy with histopathologic analysis of at least three samples for each pulmonary lobe.

Clinical data were extracted from a dedicated electronic database prospectively filled out since the epidemic outbreak in Italy on February 21, 2020. The Ethics Committee of Luigi Sacco Hospital approved the use of patients’ data for scientific research related to the disease under investigation in this study. The study followed the Italian general rules used for scientific research purposes (regulation no. 72-26/03/2012).

### Clinical–biochemical–radiological variables

The prespecified clinical, biochemical, and radiological variables were considered in the analysis.

Length of positive-pressure ventilation (PPV) was defined as continuous positive airway pressure (CPAP) *plus* mechanical ventilation (MV) lengths expressed in days. Mean positive end-expiratory pressure (PEEP) was calculated as the sum of the highest PEEP used each day divided by the number of considered days on PPV. We considered both mean PEEP during the first seven days of PPV (mPEEP_7D_) and mean PEEP during the whole hospital stay (mPEEP_TOT_). Moreover, since not only the PEEP level but also the duration of exposure to PEEP treatment may have a cumulative effect on histopathologic findings, we considered two other composite variables: cumulative PEEP defined as the arithmetic sum of daily PEEP over days on PPV (cPEEP_PPV_) and days on MV (cPEEP_MV_).

The Sequential Organ Failure Assessment score (SOFA score) was calculated for each patient to estimate the degree of organ failure (with specific scoring for neurologic, respiratory, cardiovascular, kidney, liver, coagulation dysfunction) [14]. For the data analysis, the worst value within 48 h since admission to specialized COVID-19 wards was considered. As concerns the arterial blood gas (ABG) analysis, we considered the worst ratio of arterial pressure of oxygen to inspired fraction of oxygen (pO_2_ to FiO_2_ ratio) in the first 48 h. We took the corresponding ABG values for data analysis. Regarding interleukin 6 (IL-6) and ferritin, the first available value within 1 week from admission was considered. Kidney dysfunction was evaluated according to the KDIGO classification.

In the sensitivity analysis, we considered the worst value of serum albumin, D-dimer, kidney dysfunction, and occurrence of sepsis during the hospital stay until a withholding or withdrawing decision was taken.

The degree of radiological involvement was defined on chest X-ray (CXR) at specialized ward admission according to a scoring system specifically designed for semiquantitative assessment of lung disease in COVID-19 (named Brixia score) [15]. This ranks the pulmonary involvement on an 18-point severity scale according to the extent and characteristics of lung infiltrates.

A complete list of all the variables considered for the clinical–histopathologic correlation is shown in Additional file 1.

### Autopsies and tissue processing

All autopsies were conducted between 24 and 72 h after death. Full thoracic and abdominal autopsies were performed in all cases. Lungs were removed *en bloc,* then they were macroscopically examined, and three samples of approximately 2.5 × 2 × 0.3 cm from representative areas of each pulmonary lobe (upper right, middle right, lower right, upper left, lower left) were taken and put in a 10% neutral-buffered formaldehyde for a minimum of 48 h. For each pulmonary lobe, the three areas were chosen in order to provide a representative yet comprehensive sampling, which included all the different pathological areas present in a given lobe. The specimens underwent processing with Donatello Series 2 (DiaPath, Italy) and were then embedded in paraffin. For every tissue block, a 4-µm-thick section was then cut with a microtome and stained with hematoxylin and eosin. All autopsies were conducted in compliance with biosafety recommendations of national and international regulatory agencies, including the Italian Ministry of Health, the European Centre for Disease Prevention, and the World Health Organization.

### Histopathologic patterns

Histopathologic examination was performed by two pathologists simultaneously. The following parameters were evaluated in each pulmonary lobe: (1) exudative DAD, characterized by edema with hyaline membrane formation; (2) early-phase proliferative DAD, with prominent type-II pneumocyte hyperplasia and atypia; (3) late-phase proliferative DAD, with prominent fibroblast proliferation; (4) acute fibrinous organizing pneumonia (AFOP), characterized by intra-alveolar fibrin aggregates with inflammatory cells; (5) interstitial pneumonia, with lymphocytic infiltrates within alveolar septa and interstitium; (6) bronchopneumonia, with foci of granulocytic infiltrates with or without necrosis; (7) arteriolar thrombi, with fibrin thrombi within arteriolar blood vessels; (8) intracapillary megakaryocytes; and (9) areas of normal lung (pulmonary parenchyma without features of DAD, pneumonia, and other pathological findings).

The histopathologic patterns were scored in a semiquantitative manner, based on the presence and extent of the histological findings, represented by the percentage of the tissue involved, as 0 (absent, 0%), 1 (focal/mild, 1–33%), 2 (multi-focal/moderate, 34–66%), and 3 (diffuse/extensive, 67–100%), as previously described [4].

Arteriolar thrombi were scored based on the numerosity and distribution in the three slides of each pulmonary lobe after a 10× screening of each slide, as 0 (absence), 1 (presence of 1 thrombus in 1 slide, or 2 thrombi in 2 different slides), 2 (presence of 2–3 thrombi in the same slide, or one thrombus in all three slides), or 3 (presence of > 4 thrombi in a single slide, or > 2 thrombi in 2–3 slides).

Intracapillary megakaryocytes were scored based on their numerosity in 25 high-power fields (HPF) (40×) in the most representative area identified at scanning magnification, as 0 (absence of megakaryocytes), 1 (1–5 megakaryocytes/25 HPF), 2 (6–12 megakaryocytes/25 HPF), or 3 (≥ 13 megakaryocytes/25 HPF) [16].

According to the scoring system, the total score of each histopathologic pattern ranges from 0 to 15.

Additional findings were also recorded, including areas of infarct, hemorrhage, fibrosis, pleuritis; isolated edema (without hyaline membrane formation); the presence of intra-alveolar lymphocytes, plasma cells, giant cells; and Aspergillus hyphae.

### Statistical analysis

For continuous variables, the data are shown as median (IQR). The normal distribution was checked using the Kolmogorov–Smirnov test, and the equality of variances was checked with Levene’s test. Differences between two groups of continuous variables were tested with Student’s *t* test or with Mann–Whitney test, as appropriate. The analysis of differences between three or more groups was performed with one-way analysis of variance (ANOVA), or Kruskal–Wallis test, followed by post hoc Tukey's or Dunn's test for multiple comparisons, as appropriate. Correlation coefficients between continuous variables were assessed with Spearman's correlation. Differences in categorical variables were analyzed with Fisher's exact test. Comparison of survival curves was analyzed with the log rank Mantel–Cox test.

Moreover, we applied an agglomerative hierarchical clustering using the nine prespecified histopathologic patterns. This method has been previously used in critically ill patients [17, 18]. It builds homogeneous clusters based on dissimilarities or distances between cases and then proceeds iteratively to join the most similar cases. All the nine features used had the same scale ranging from 0 to 15; therefore, values were not further scaled. The "Euclidean" distance was computed among observations, and a complete linkage was used to set the distance among the clusters. The process was visualized using dendrograms, and the cut-off value for cluster identification was set in order to identify up to five clusters [19, 20]. The optimal number of clusters was chosen according to the “elbow” method [21] and the consensus of the researchers. The hierarchical model was built considering all complete lung autopsies.

Finally, a multiple variable analysis was performed by fitting a generalized linear model including the relevant variables, which were significant at the univariate analysis, as covariates.

Statistical analysis was carried out using SPSS 27 (IBM, Armonk, NY) and R version 4.0.3 (R Foundation for Statistical Computing, Vienna, Austria). A *p* < 0.05 was considered significant for two-tail tests.

## Results

In the study period, 92 complete autopsies (24 females and 68 males) were conducted at our center. They represent all the autopsies carried out at the Pathology Unit of L. Sacco Hospital from February 29 to June 30, 2020. The flowchart of the studied population is summarized in Additional file 1: Fig. S1.

The full pulmonary examination was available only in 75 patients; in the remaining 17 cases, it was not possible to carry out a complete macroscopic examination of the lungs due to sampling problems. These cases were removed from the study. Forty-eight patients (64%), 44 from ICU (91.7%) and four (8.3%) from the medical ward, had complete clinical records. Among the patients without complete clinical data (*n* = 27), three (11.1%) died at home, six (22.2%) died in the emergency department, and 18 (66.7%) died in other hospitals (nine in ICU, eight in medical wards, one missing data). The prevalence of patients admitted to ICU was different between groups with and without clinical data (91.7% vs. 33.3%, *p* < 0.0001).

Clinical characteristics, main laboratory, and radiological findings are shown in Table 1 and Additional file 1: Table S1.

**Table 1:** Clinical–biochemical–radiological characteristics of patients included in the final data analysis

	*N* = 48
Age, years	64.5 (60–71)
BMI	27.7 (24.5–31.1)
Gender, M/F, *n* (%)	44/4 (91.7/8.3)
Comorbidities, *n* (%)	
Smoke	9 (18.8)
Cardiovascular disease	30 (62.5)
Respiratory disease	3 (6.3)
Immunosuppressive therapy	2 (4.2)
HIV	1 (2.1)
Diabetes	5 (10.4)
Cancer	6 (12.5)
Symptoms onset-to-hospital admission interval, days	7 (4–10)
Symptoms onset-to-mechanical ventilation, days	12 (8.7–17.5)
Chest X-ray score^a^	15 (13–16)
CPAP treatment, *n* (%)	39 (81.2)
CPAP days	2 (1–5)
Patients treated with MV, *n* (%)	43 (89.6)
MV days	15 (7–19)
PPV days	17.5 (9–23.7)
FiO_2_	0.8 (0.7–0.8)
PEEP, cmH_2_O	14 (12–17.5)
mPEEP_TOT_, cmH_2_O	13.6 (10.5–16)
cPEEP, cmH_2_O·days	193.4 (123.4–279.5)
Ppeak, cmH_2_O	33 (30–35)
Tidal volume, ml	550 (500–600)
PaO_2_/FiO_2_ ratio	116.5 (93.7–142.5)
ABG	
pH	7.33 (7.27–7.4)
pCO_2_, mmHg	46.5 (40–54.5)
pO_2_, mmHg	86.5 (70.5–101.2)
Prone position, *n* (%)	10 (20.8)
Mean arterial pressure, mmHg	75 (70–80)
Norepinephrine, *n* (%)	31 (64.6)
Fluid balance, ml	1025 (177–1817)
AKI, *n* (%)	
None	11 (22.9)
KDIGO class I	11 (22.9)
KDIGO class II	4 (8.3)
KDIGO class III	22 (45.8)
CRRT, *n* (%)	11 (22.9)
Neutrophils, cells·mm^3^	8340 (5060–11,000)
Lymphocytes, cells·mm^3^	536 (413–815)
Neutrophils-to-lymphocytes ratio	16.3 (7.7–23.1)
Hemoglobin, g dl^−1^	12.7 (11.5–13.8)
Hematocrit, %	37.5 (35–41)
Platelets, 10^3^ cells·mm^3^	217.5 (174–331)
C-reactive protein, mg l^−1^	220 (126–322)
Procalcitonin, µg l^−1^	1.25 (0.27–2.6)
Worst S-albumin, g dl^−1^	1.7 (1.6–2)
Worst D-dimer, ng ml^−1^	13,390 (3758–29,524)
Fibrinogen, mg ml^−1^	700 (695–700)
IL-6, ng l^−1^	254 (160–1047)
Ferritin, µg l^−1^	2560 (1282–3494)
S-creatinine, mg dl^−1^	0.95 (0.78–1.41)
LDH, U l^−1^	543 (437.5–703.7)
SOFA score	10 (8–11.75)
Pharmacological treatment, *n* (%)	
Tocilizumab	12 (25)
Remdesivir	11 (22.9)
Hydroxychloroquine	31 (64.6)
Lopinavir/ritonavir	26 (54.2)
Steroids	14 (29.2)
Sepsis/septic shock, *n* (%)	28 (58.3)
LOS—ICU, days	15 (6.25–19.75)
LOS—hospital, days	17.5 (11–25)

Data are shown as median (IQR) or *n* (%) were indicated. A complete list of variables is shown in Additional file 1: Table S1

*BMI* body mass index, *CPAP* continuous positive airway pressure, *MV* mechanical ventilation, *PPV* positive-pressure ventilation, *PEEP* positive end-expiratory pressure, *mPEEP*_*TOT*_ mean PEEP applied with PPV during hospitalization, *cPEEP* cumulative PEEP, *Ppeak* peak airway pressure measured at admission in mechanically ventilated patients, *ABG* arterial blood gas analysis, *AKI* acute kidney injury, *CRRT* continuous renal replacement therapy, *IL-6* plasma interleukin 6, *LDH* lactic dehydrogenase, *SOFA* Sequential Organ Failure Assessment, *LOS* length of stay, *ICU* intensive care unit

^a^Chest X-ray score according to Brixia classification

**Fig. 1:**
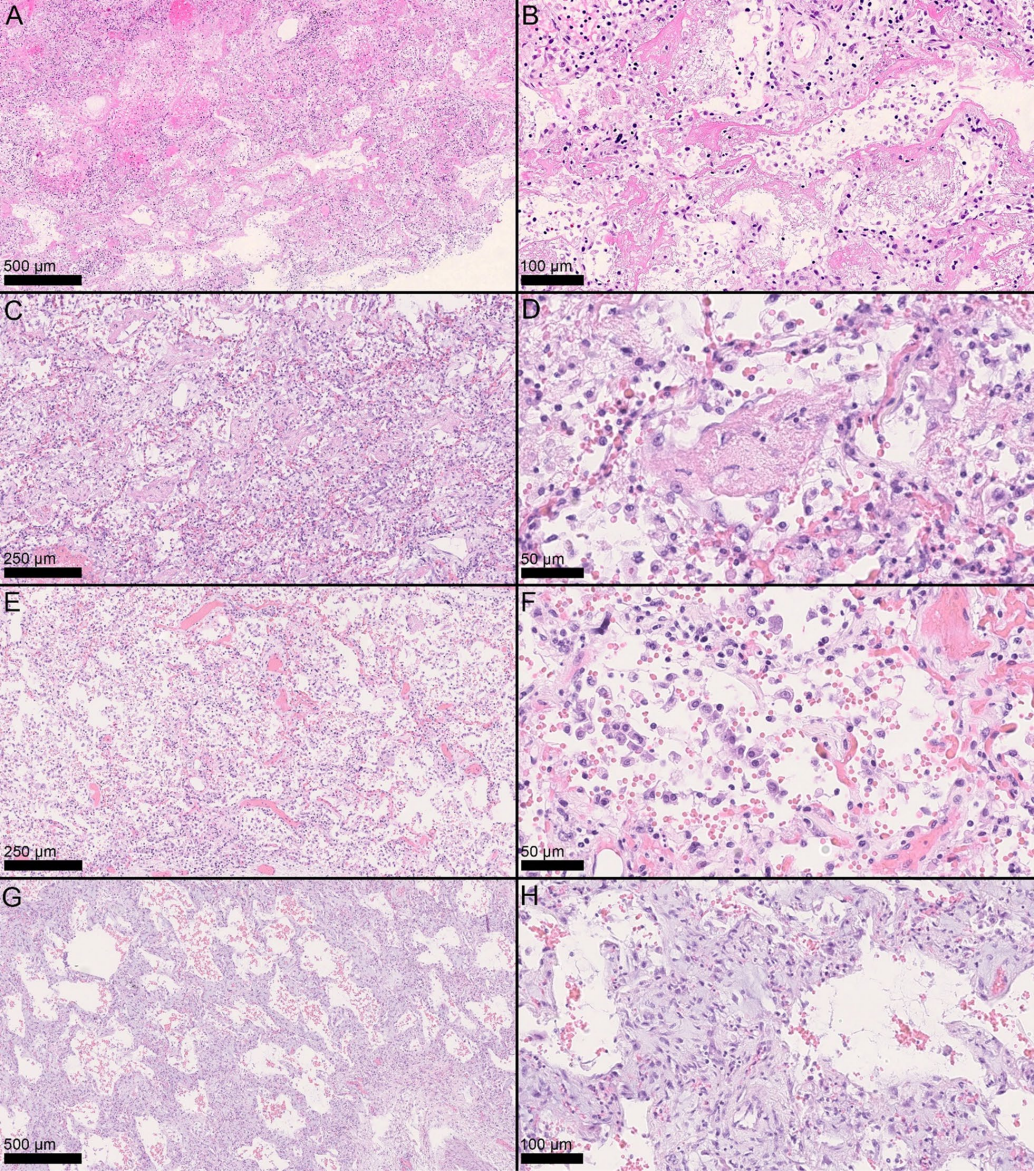
Histopathologic patterns and findings. Exudative phase DAD, with hyaline membranes and edema (**A** H&E, 5×; **B** H&E, 20×). Acute fibrinous organizing pneumonia (AFOP) with fibrinous endoalveolar aggregates encompassing inflammatory cells (**C** H&E, 10×; **D** H&E, 40×). Early proliferative phase DAD with hypercellularity, type II pneumocyte hyperplasia and atypia (**E** H&E, 10×; **F** H&E, 40). Late proliferative phase DAD with interstitial fibroblast proliferation (**G** H&E, 5×; **H** H&E, 20×)

**Fig. 2:**
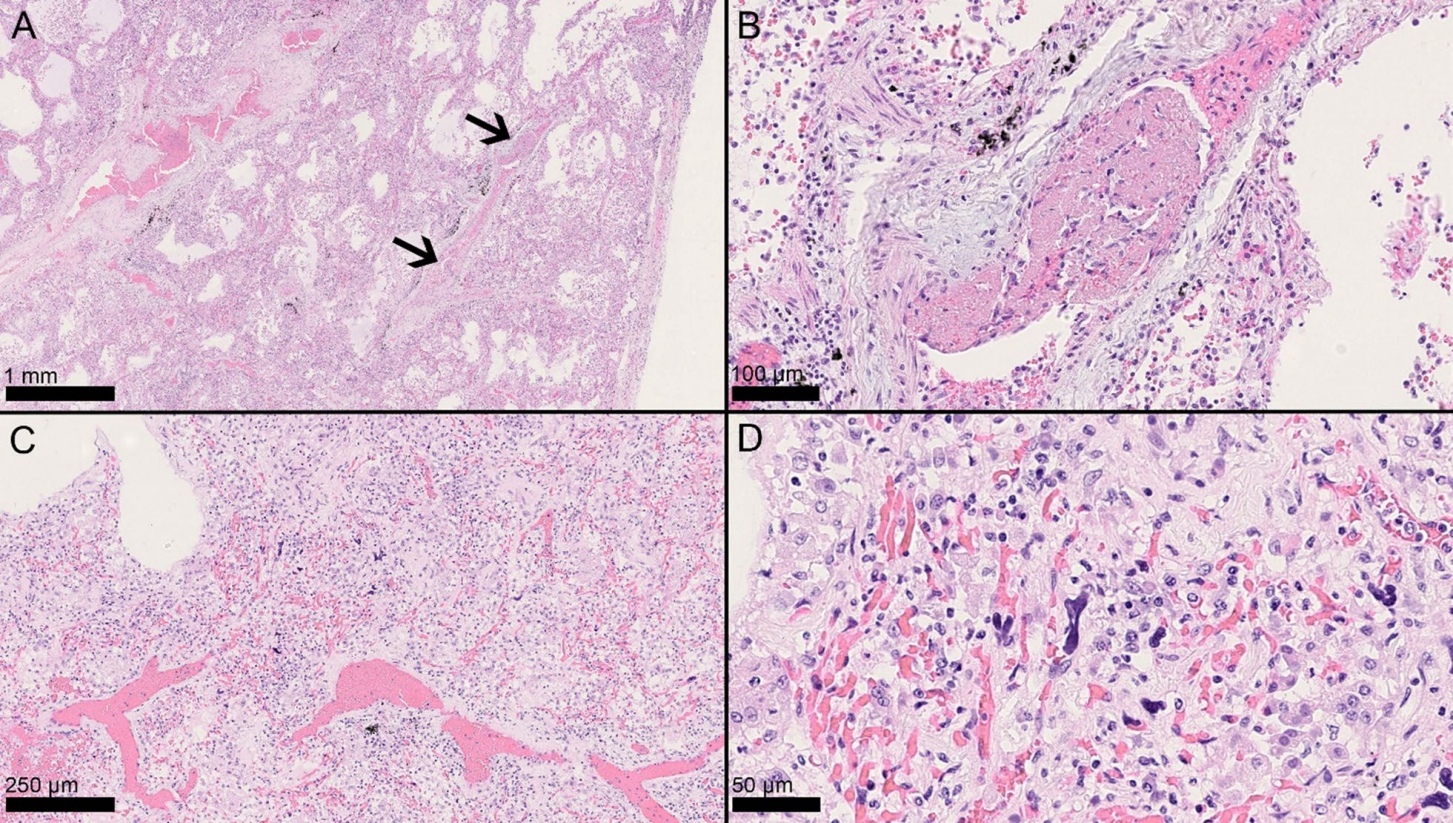
Histopathologic patterns and findings. Microthrombi and megakaryocytes in diffuse alveolar damage (DAD). Two microthrombi within the same arterial vessel in the setting of vascular congestion (**A**, arrows, H&E, 2.5×), composed of fibrin, erythrocytes, and inflammatory cells (**B**, H&E, 20×). Increased intracapillary megakaryocytes were sometimes evident even at low power (**C**, H&E, 10×) and could be readily identified at high power due to their characteristic hyperchromatic, branching nuclei confined within capillary vessels (**D**, H&E, 20×)

Macroscopically, the lungs showed a spectrum of alterations ranging from complete consolidation to near-normal parenchyma; most frequently, a given pulmonary lobe presented areas with different degrees of consolidation. Main histopathologic findings are shown in Figs. 1 and 2 and are summarized in Additional file 1: Table S2.

**Fig. 3:**
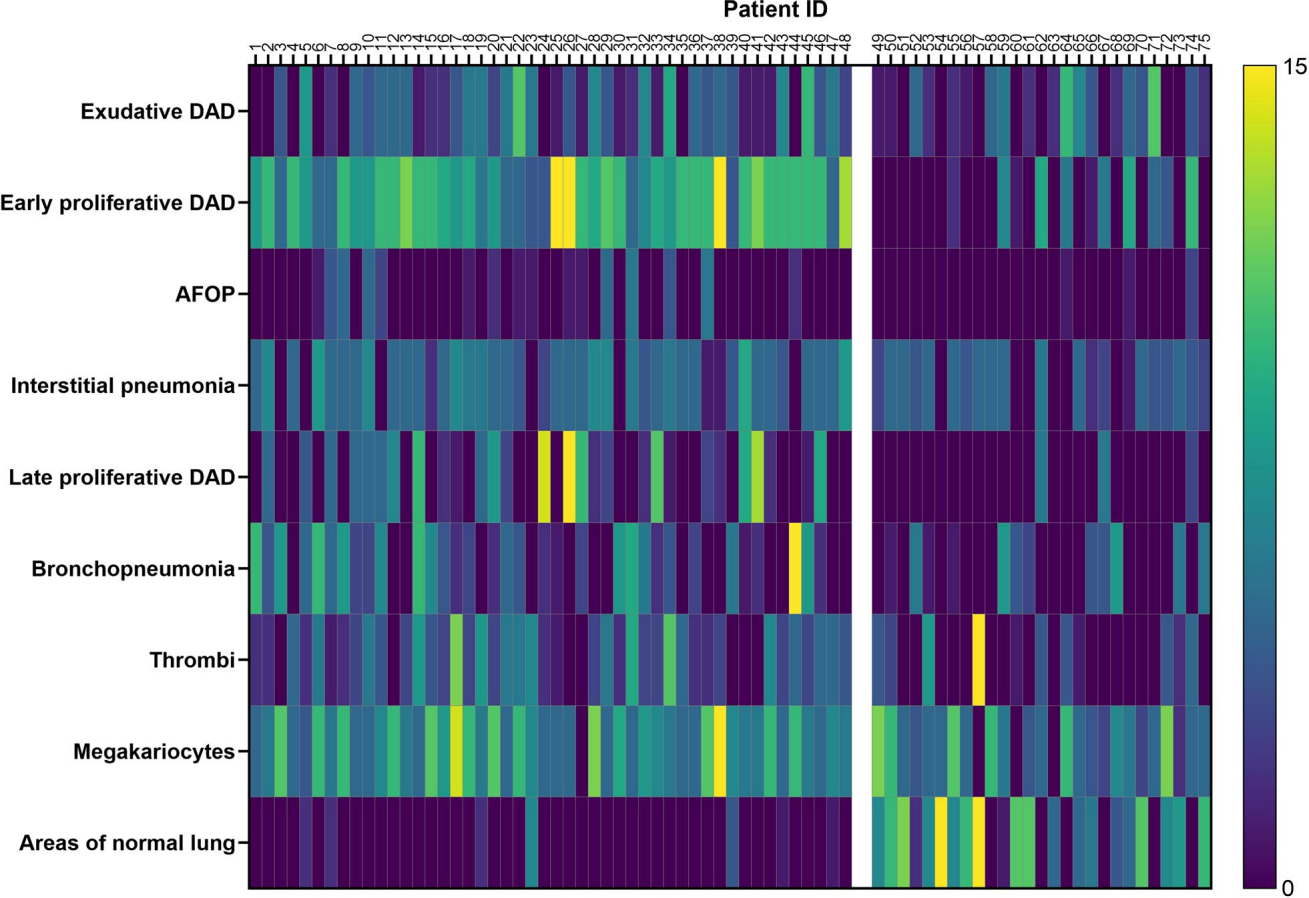
Heatmap of histopathologic patterns of the studied subjects. Patients on the right (*n* = 27) had no full clinical data: 18 came from other hospitals, three came directly from home, and six were emergency department patients. They differed from patients on the left in the extension of early proliferative DAD, AFOP, interstitial pneumonia, late proliferative DAD, bronchopneumonia, thrombi, megakaryocytes, and normal lung areas (see Additional file 1: Table S2). Only nine patients in the group on the right were admitted to ICU; the remaining died in medical wards or ED. Although in the group on the right clinical data are missing, the exposure to PPV and PEEP is presumptively different from the patients considered in the analysis (group on the left) on which 43 out of 48 subjects underwent to mechanical ventilation. The column on the right shows the histopathologic score (see methods in the text)

The extent and coexistence of patterns in each studied subject of the whole cohort are shown in Fig. 3.

The correlation analysis between clinical characteristics and histopathologic findings was conducted on 48 patients with available complete clinical records.

Exudative DAD had a weak correlation with length of stay (LOS) in hospital (*r* = − 0.399, *p* = 0.005), MV days (*r* = –0.304, *p* = 0.048), and PPVdays (*r* = − 0.35, *p* = 0.015). AFOP was revealed to have a weak correlation with LOS in ICU (*r* = 0.363, *p* = 0.015), MVdays (*r* = 0.353, *p* = 0.02), procalcitonin (*r* = 0.404, *p* = 0.005), worst serum albumin (*r* = − 0.335, *p* = 0.021), ferritin (*r* = 0.36, *p* = 0.034), total SOFA (*r* = 0.345, *p* = 0.016), and cPEEP_MV_ (*r* = 0.365, *p* = 0.017). Late proliferative DAD showed a correlation with LOS in hospital (*r* = 0.5, *p* < 0.001), LOS in ICU (*r* = 0.39, *p* = 0.009), PPVdays (*r* = 0.48, *p* = 0.001), MVdays (*r* = 0.48, *p* = 0.001), WBC count (*r* = 0.33, *p* = 0.022), cPEEP_PPV_ (*r* = 0.42, *p* = 0.004), and cPEEP_MV_ (*r* = 0.426, *p* = 0.004).

No significant correlation was found between clinical–biochemical–radiological parameters and early proliferative DAD or bronchopneumonia.

Thrombi had a positive correlation with CXR score (*r* = 0.409, *p* = 0.004) and IL-6 (*r* = 0.362, *p* = 0.049). Areas of normal lung were correlated with LOS in hospital (*r* = − 0.3, *p* = 0.038), PPVdays (*r* = − 0.369, *p* = 0.01), baseline serum albumin (*r* = 0.312, *p* = 0.031), worst serum albumin (*r* = 0.43, *p* = 0.008), cPEEP_PPV_ (*r* = − 0.373, *p* = 0.011), and cPEEP_MV_ (*r* = − 0.373, *p* = 0.015).

There was no correlation between histopathologic findings and intervals from (1) symptoms onset to hospitalization, (2) first non-respiratory symptoms to dyspnea onset, (3) CPAP to mechanical ventilation start, and (4) symptoms onset to mechanical ventilation start (Additional file 1: Fig. S2).

**Fig. 4:**
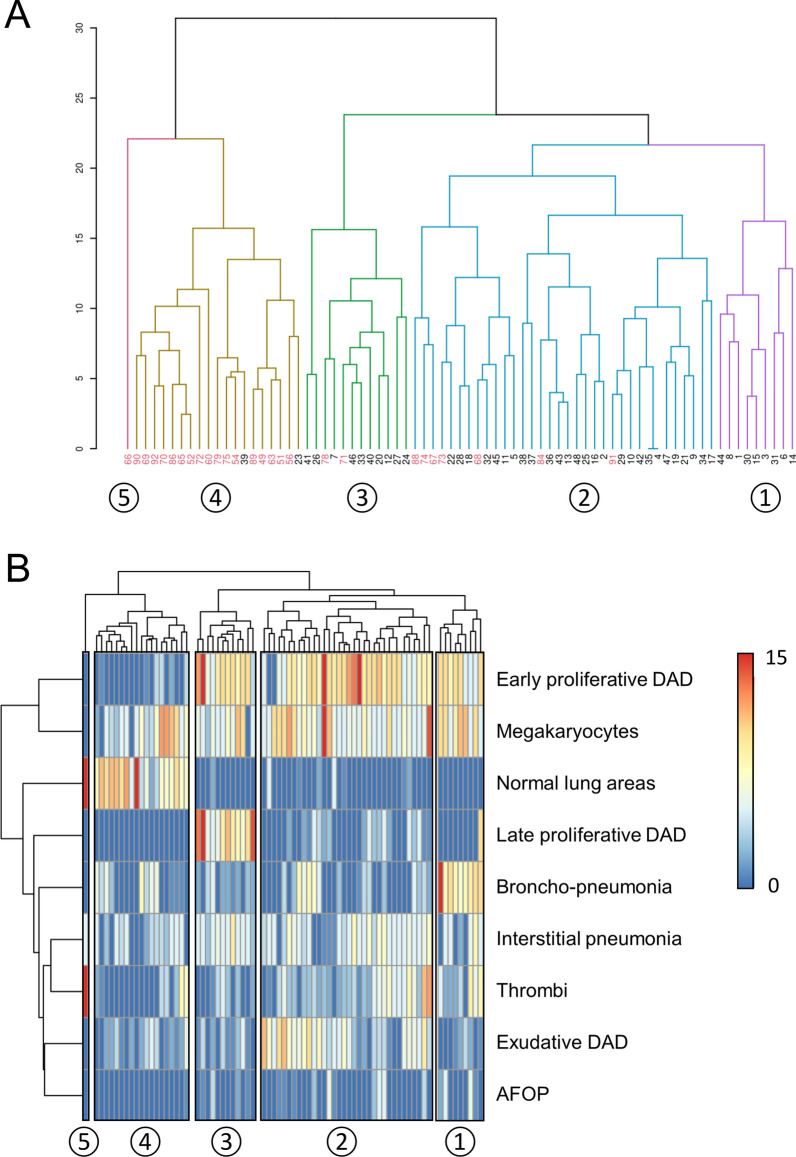
Cluster analysis. **A** The dendrogram of hierarchical clustering. Patients with clinical data are labeled in black at the bottom of the dendrogram. **B** The characteristics of histopathologic patterns according to the clustering. On the left, the color scale according to the semiquantitative histopathologic assessment is shown. Only two patients of cluster 4 and none of cluster 5 had clinical data. Overall, patients with no clinical data (*n* = 27) had a short LOS in hospital because three died at home and six shortly after emergency department access. Moreover, only nine of them (33.3%) were admitted to ICU and underwent mechanical ventilation. Cluster labels are indicated by the circled numbers

Five clusters were identified in the whole cohort of 75 autoptic examinations, and four clusters were also represented in the cohort of 48 patients with complete clinical data (Fig. 4a). The optimal number of clusters is shown in Additional file 1: Fig. S3. Cluster 4 consisted mainly of patients without clinical data in whom areas of normal lung were predominant. The main histopathologic features of different clusters are summarized in Table 2. As shown in Fig. 4b, cluster 3 was characterized by a high prevalence of both early DAD and late proliferative DAD compared to the other clusters.

**Table 2:** Histopathologic features of different clusters

	Cluster 1 *N* = 9	Cluster 2 *N* = 34	Cluster 3 *N* = 12	Cluster 4 *N* = 19	Cluster 5 *N* = 1	*p* value*
Early proliferative DAD	10 (5–10)	9 (7–10)	9.5 (7.5–10)	0 (0–1)	0	< 0.001
Megakaryocytes	10 (5–10)	6 (5–9.5)	5.5 (5–6.25)	5 (4–7)	0	0.076
Normal lung areas	0 (0–0)	0 (0–0)	0 (0–0)	7 (6.5–10.5)	15	< 0.001
Late proliferative DAD	0 (0–0)	1 (0–3)	9 (6.75–11.5)	0 (0–0)	0	< 0.001
Bronchopneumonia	9 (8–10)	0 (0–4)	2 (0.75–2.25)	1 (0–5)	0	< 0.001
Interstitial pneumonia	5 (0–5)	5 (4–5.75)	5 (5–5)	4 (0–5)	5	0.017
Thrombi	4 (2–6)	3 (3–5)	0.5 (0–3)	0 (0–2.5)	15	0.003
Exudative DAD	1 (0–2)	5 (4–7)	1 (0.75–3.25)	2 (0.5–3)	0	< 0.001
AFOP	0 (0–2)	0 (0–1)	0 (0–1)	0 (0–0)	0	0.085

Data are shown as median (IQR)

^*^With the exclusion of cluster 5

**Fig. 5:**
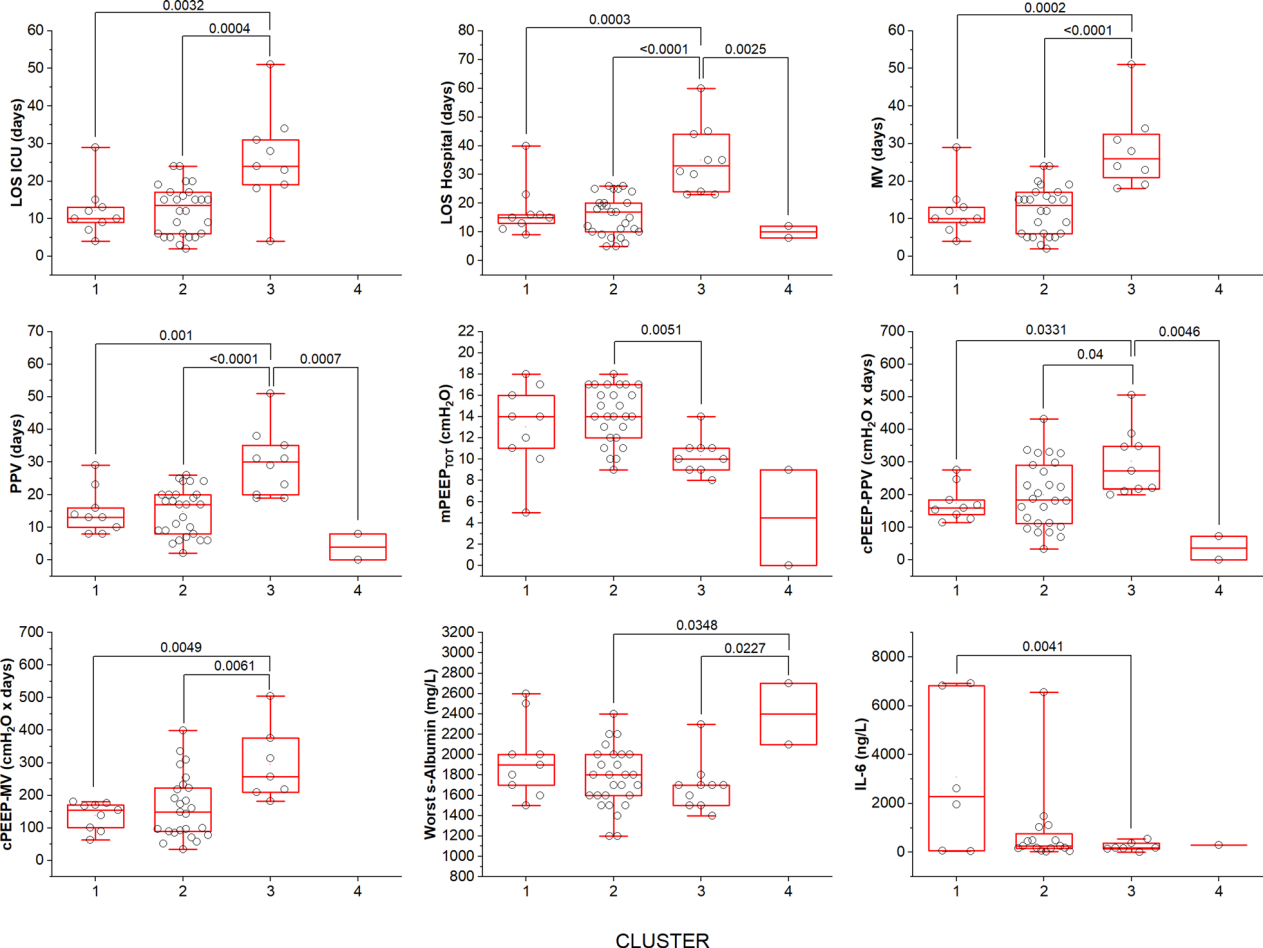
Distributions of the means according to clusters (*n* = 48). Whiskers represent the range. Cluster 4 mainly consisted of patients without clinical data. None of them was admitted to ICU nor underwent mechanical ventilation

**Fig. 6:**
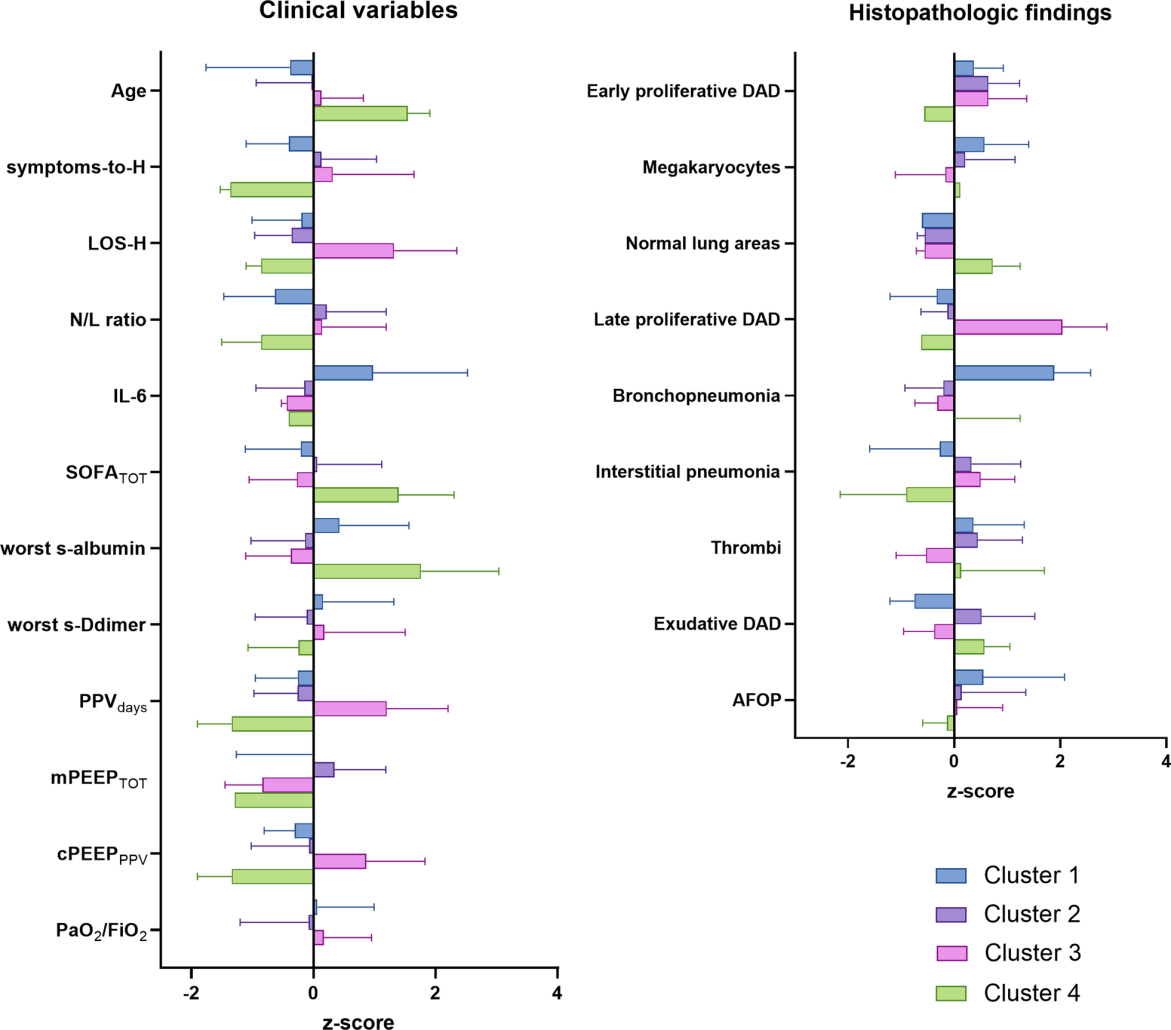
Clinical (left) and histopathologic (right) characteristics of the clusters. Continuous variables were z-transformed (each distribution has mean of 0 and standard deviation of 1) and then plotted to a common axis. Values are shown as means and SD. LOS-H, length of stay in hospital; PPV_days_, days on positive-pressure ventilation (CPAP and/or mechanical ventilation); mPEEP_TOT_, mean positive end-expiratory pressure during PPV; cPEEP_PPV_, cumulative PEEP, during PPV; symptoms-to-H, interval between symptoms onset and hospital admission; N/L ratio, ratio between neutrophils and lymphocytes at specialized ward admission

LOS in hospital (*p* < 0.0001), LOS in ICU (*p* < 0.0001), PPVdays (*p* < 0.0001), mPEEP_TOT_ (*p* = 0.007), MVdays (*p* < 0.0001), cPEEP_PPV_ (*p* = 0.003), cPEEP_MV_ (*p* = 0.003), worst serum albumin (*p* = 0.017), IL-6 (*p* = 0.047), and Kidney SOFA (*p* = 0.001) were different among clusters (Fig. 5). The major differences were detected in the duration of hospitalization and positive-pressure ventilation (PPV or MV) between cluster 3 and the others. Intervals from (1) symptoms onset to hospitalization, (2) first non-respiratory symptoms to dyspnea onset, (3) CPAP to mechanical ventilation start, (4) symptoms onset to mechanical ventilation start did not differ between clusters (Additional file 1: Fig. S4). An overview of histopathologic and clinical characteristics of clusters is shown in Fig. 6.

Among the pharmacological treatments shown in Table 1, only steroid use was associated with cluster 3 (*p* = 0.04). Moreover, a history of cardiovascular disease was also associated with cluster 3 (*p* = 0.023). At the multiple variable analysis, age, worst serum albumin, mPEEP_TOT_, and PPVdays were fitted into the generalized linear model as continuous covariates, steroid treatment as binomial covariate, and cluster 3 (yes/no) as dependent variable. As a result, only mPEEP_TOT_ and PPVdays, with interaction effect, were significant (Table 3).

**Table 3:** Multiple variable analysis

Parameter	CI95% (*β*)			
	*β*	Lower	Upper	*p* value
Age	1.173	0.959	1.434	0.12
Worst s-albumin	0.998	0.993	1.004	0.508
Steroid therapy	0.274	0.035	2.123	0.21
mPEEP_TOT_ * PPVdays	1.015	1.001	1.03	0.04

Dependent variable: Cluster 3. mPEEP_TOT_ and PPVdays were considered with interaction effect because they are highly correlated with each other

**Fig. 7:**
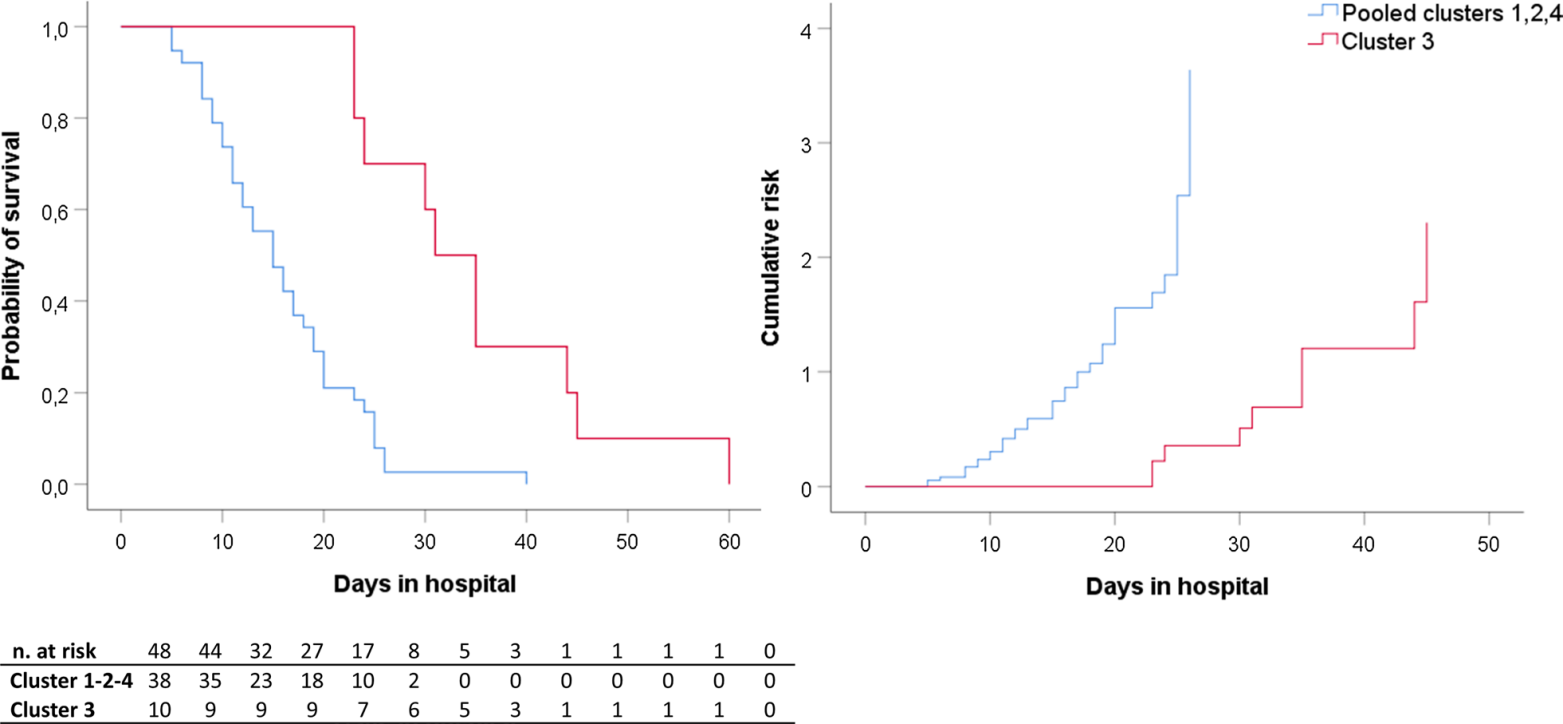
Survival analysis. Kaplan–Meier curves of clusters of patients with full clinical records. Cluster 3 vs pooled clusters 1, 2, and 4, *p* < 0.0001

Survival curves were different between cluster 3 and the other clusters (LOS in hospital 33 [25.5–41.7] vs. 15 [10.2–20] days, *p* < 0.0001) (Fig. 7). Kaplan–Meier curves of clusters are shown in detail in Additional file 1: Figs. S5 and S6.

Finally, five patients on 75 autopsies also had invasive aspergillosis (Additional file 1: Fig. S7).

## Discussion

The contribution of postmortem studies to our knowledge of the pathology underlying the clinical picture in COVID-19 is undeniable, and the topic is still under investigation. We found that some clinical parameters and, of particular interest, the long-lasting application of positive pressure to the patients’ airways are correlated with specific clustered histopathologic findings. Interestingly, the intensity and the duration of positive-pressure ventilation were associated both with exudative DAD and late proliferative DAD; consistently, a negative correlation of the same parameters with areas of normal lung has been found.

Since the first case series, efforts have been made to gather available studies and try to elaborate hypotheses on potential unifying pathophysiological mechanisms [22]. However, most of these studies focus on pathology and suffer from the lack of data on clinical-laboratory and imaging findings, so that they do not allow to get insights or at least valuable inputs on their possible links.

Indeed, confounding factors may be several in such a complex clinical scenario, and detecting straightforward causal relationships, discriminating the net impact of each player, seems impossible. Although different histopathologic findings are known to vary according to the time course of COVID-19 progression, our results suggest that temporality is not the only (and possibly not even the main) factor that shows a correlation with the development of lung injury. In fact, our results highlight that it is possible to discriminate different clinical courses (thus including treatments applied), which are associated with a variable mix of histopathologic findings, probably due to the impact of a variable mix of complex underlying pathophysiological mechanisms, often acting simultaneously, with a changing degree and duration.

In particular, the development of late proliferative DAD, which is characterized by collagen deposition, prevails in cluster 3, and it is correlated with prolonged treatment with positive-pressure ventilation.

It has been demonstrated that SARS-CoV-2 directly damages both endothelial and epithelial cells in lung tissue [23]. Our group also described damage of both type II pneumocytes and endothelial cells, with intracytoplasmic virions and disruption of adherens junctions, in patients who died for COVID-19 [24]. Our study suggests that even the length of exposure to physical stressors, such as prolonged use of positive pressure, correlates with advanced DAD stages with fibroproliferative features and markedly reduced normal areas.

Interestingly, the significant correlation between serum albumin and both AFOP and areas of normal lung corroborates the likely link between hypoalbuminemia and alterations of endothelial permeability [24].

The correlation between thrombi and chest X-ray score as well as IL-6 values reveals that patients with more compromised interstitial patterns and more pronounced inflammatory response are at higher risk of developing thrombotic complications, in line with the "immunothrombosis" hypothesis [11, 25].

Severe COVID-19 is characterized by a marked hyperinflammatory response resulting in a cytokine storm, with dysregulated production of proinflammatory cytokines (e.g., IL-1β and IL-6), resulting in significant lymphopenia, C-reactive protein increase, and a profound vascular dysfunction. Both direct and immune-mediated endothelial injury may act as procoagulant triggers, resulting in hypercoagulability and thromboinflammation, which lead to intra-alveolar activation of coagulation and thrombin generation [26]. The extensive endothelial damage is marked by increased D-dimer levels and increased incidence of venous thrombosis and pulmonary thromboembolism [27]. In fact, postmortem findings of COVID-19 patients revealed diffuse alveolar damage with severe capillary congestion, thrombosis of pulmonary capillaries, small arteries < 1 mm, midsized arteries, and pulmonary thromboembolism suggesting systemic endothelial dysfunction [4, 28]. These features may explain the peculiar mechanoelastic properties of the respiratory system in COVID-19 ARDS, which at presentation is characterized by higher compliance than other kinds of ARDS [29]. Interestingly, we did not find any significant correlation between peak airway pressure or elevated D-dimer and C-reactive protein levels and specific histopathologic findings in our series. However, cumulative PEEP during the hospital stay was positively correlated with late proliferative DAD and it was higher in cluster 3, which was characterized by prevailing early and late proliferative DAD.

These findings could be explained by the relevance not only of the inflammatory (and procoagulant) macro- and microenvironment per se at baseline, but also of the prolonged interplay of such inflammatory factors with protracted exposure to positive pressures and mechanical ventilation (the latter carrying benefits but also potentially harmful effects on lung tissues).

It is intriguing that patients who died soon after the onset of respiratory failure showed a trend to more diffuse areas of normal lung, probably due to less prolonged exposure to the cytokine storm and therapeutic interventions such as positive-pressure and mechanical ventilation. The significantly lower representation of proliferative DAD in these rapidly worsening patients is consistent with results obtained from antemortem lung biopsies [30]. Furthermore, we found a positive correlation between steroid use and cluster 3 at the univariate analysis. This result is unexpected and elicits several considerations and hypotheses: A selection bias cannot be excluded. During the first pandemic wave, this treatment did not still represent the “standard of care” and was often administered as rescue therapy in very compromised patients. Moreover, the low fraction of patients who received steroids (29.2%) and a non-standardized use of such therapy (as regards timing and dose) during the first COVID-19 outbreak should be taken into consideration when interpreting these correlations. Therefore, these results require further confirmation.

Clustering allowed us to highlight the correlation between clinical variables and complex histological pictures deriving from a variable mix of each pattern (e.g., overlap between exudative DAD and features of AFOP), which contributes to the severe distortion of lung architecture characterizing COVID-19.

This study has some limitations. Our findings derive from autopsies conducted during the first pandemic wave in Italy, when steroid use was inconstant and probably delayed in the course of the disease. However, the proportion of patients undergoing such a treatment strategy is not-negligible, and some patients (25%) who were not administered steroid treatment received tocilizumab, a therapeutic strategy that has been a matter of debate in severe COVID-19 and whose impact on lung tissues is still only partially understood. Nonetheless, we believe that we actually had the rare and precious opportunity to observe the clinical course of a new condition, with only a few confounding factors, which might have limited or blunted the complex inflammatory mechanisms which lead to initiation and development of the clinical–biochemical–radiological and histopathologic picture.

The choice of the optimal number of clusters is usually a difficult task. This is a crucial issue which has been matter of debate in the literature, and it is undeniable that the process of clustering may carry a certain degree of arbitrariness. However, when used with awareness of the source of data to be analyzed and their clinical meaning and value, clustering can be a powerful statistical tool, which allows to interpret the complexity of the clinical picture. In our study clustering resulted to be informative: The association between clusters and clinical features was strong and explained by plausible pathophysiological mechanisms.

Finally, our findings are based on postmortem findings: The histopathologic patterns of survivors (and possibly their correlation to the clinical picture) may show some dissimilarities from those of non-survivors.

## Conclusions

Our results provide novel insights into the correlations between the clinical–biochemical–radiological picture and the histopathologic findings in patients with severe COVID-19. Therefore, they may prove helpful in deepening our awareness of the disease course, suggesting clues to the complexity of the underlying pathophysiological mechanisms.

## Supplementary Information

**Additional file 1.** List of clinical-laboratory variables considered in the statistical analysis. **Table S1.** Clinical-biochemical-radiological characteristics of patients included in the final data analysis. **Table S2.** Histopathologic findings. **Fig. S1.** Flow chart of the patient selection. **Fig. S2.** Correlation matrix. **Fig. S3.** Choice of the number of clusters (K). **Fig. S4.** Distribution of intervals from symptoms onset to hospital admission, from symptoms to dispnoea onset, from symptoms onset to MV start, and from CPAP to MV start among clusters. **Fig. S5.** Kaplan Meier curves of all analyzed patients. **Fig. S6.** Kaplan Meier curves of clusters of patients treated with positive pressure ventilation. **Fig. S7.** Histopathology of a case with aspergillosis.

## Data Availability

Data are available on reasonable request and after institutional ethical committee authorization.

## Abbreviations

ABG: Arterial blood gas
AFOP: Acute fibrinous organizing pneumonia
COVID-19: Coronavirus 2019 disease
CPAP: Continuous positive airway pressure
CXR: Chest X-ray
DAD: Diffuse alveolar damage
HPF: High-power fields
ICU: Intensive care unit
IL-1β: Interleukin 1β
IL-6: Interleukin 6
KDIGO: Kidney Disease Improving Global Outcomes
LOS: Length of stay
MV: Mechanical ventilation
PEEP: Positive end-expiratory pressure
mPEEP_7D_: Mean PEEP during the first seven days
mPEEP_TOT_: Mean PEEP during the whole hospital stay
cPEEP_PPV_: Cumulative PEEP over positive-pressure ventilation days
cPEEP_MV_: Cumulative PEEP over mechanical ventilation days
PPV: Positive-pressure ventilation
SOFA: Sequential Organ Failure Assessment score
