# Supplementary material for: Lung histopathologic clusters in severe COVID-19: a link between clinical picture and tissue damage
Source: Crit Care. 2021 Dec 13;25:423. doi: 10.1186/s13054-021-03846-5 (PMC8667540; doi:10.1186/s13054-021-03846-5)
Supplement: Supplementary file 1 — Additional file 1. List of clinical-laboratory variables considered in the statistical analysis. Table S1. Clinical-biochemical-radiological characteristics of patients included in the final data analysis. Table S2. Histopathologic findings. Fig. S1. Flow chart of the patient selection. Fig. S2. Correlation matrix. Fig. S3. Choice of the number of clusters (K). Fig. S4. Distribution of intervals from symptoms onset to hospital admission, from symptoms to dispnoea onset, from symptoms onset to MV start, and from CPAP to MV start among clusters. Fig. S5. Kaplan Meier curves of all analyzed patients. Fig. S6. Kaplan Meier curves of clusters of patients treated with positive pressure ventilation. Fig. S7. Histopathology of a case with aspergillosis. [file 13054_2021_3846_MOESM1_ESM.pdf]

Supplemental material

Lung histopathologic clusters in severe COVID-19: a link between clinical picture and tissue damage

Wu MA, Lopez G, Nebuloni M, Ottolina D, Montomoli J, Carsana L, Fossali T, Castelli A, Rech R, Cogliati C, Catena E, and Colombo R.

List of clinical-laboratory variables considered in the statistical analysis:

1. Age; gender; body mass index; history of diabetes, respiratory disease, smoking (active or past), cardiovascular disease, cancer, chronic immunosuppressive therapy, and HIV infection; interval between symptoms onset and hospitalization; interval between symptoms onset and dyspnea onset; length of stay in hospital (LOS-H); length of stay in ICU (LOS-ICU); days on continuous positive airway pressure (CPAP); days on mechanical ventilation; interval between CPAP and mechanical ventilation onset; days on positive pressure ventilation (PPV); interval between symptoms onset and mechanical ventilation; highest positive end-expiratory pressure (PEEP) in the first 48 hours since positive pressure ventilation onset; mean PEEP in the first seven days on positive pressure ventilation ($mPEEP_{7D}$); mean PEEP during whole positive pressure ventilation treatment ($mPEEP_{TOT}$); cumulative PEEP over positive pressure ventilation days ($cPEEP_{PPV}$); cumulative PEEP over mechanical ventilation days ($cPEEP_{MV}$); tracheostomy (yes/not); need for continuous renal replacement treatment (CRRT); interval between CPAP and CRRT onset; interval between mechanical ventilation and CRRT onset; treatment with Tocilizumab, Remdesivir, Hydroxychloroquine, and lopinavir/ritonavir (yes/not); need for prone position (yes/not) during hospital stay; sepsis (yes/not) during hospital stay; worst serum albumin during hospital stay; worst D-dimer during hospital stay; kidney injury during hospitalization assessed by KDIGO classification.
2. Worst value within 48 hours since internal medicine ward or ICU admission of the following variables: Daily urinary output; daily fluid balance; Glasgow Coma Score, ratio of oxygen arterial pressure to fraction of inspired oxygen (paO_2/FiO_2) and the corresponding values of pH, pO_2, pCO_2, $SatO_2$%, plasma lactate; chest-X-ray score (assessed through Brixia score); airway peak pressure (in mechanically ventilated patients); tidal volume (in mechanically ventilated patients); respiratory rate; mean arterial pressure; need for norepinephrine, epinephrine or dobutamine infusion (and dosages); serum creatinine; white blood cell count (total and neutrophils and lymphocytes count); neutrophils to lymphocytes ratio; haemoglobin; haematocrit; platelets count; C-reactive protein;

procalcitonin; serum albumin; D-dimer; serum blood urea; serum bilirubin; serum aspartate-amino-transferase; serum alanine-amino transferase; serum lactate-dehydrogenase; serum cholesterol (total and HDL and LDL partitions); serum phosphate; serum magnesium, calcium, sodium, potassium and chloride; sequential organ failure assessment (SOFA) score. First available value of interleukin-6 and ferritin within one week since hospital admission.

e-Table 1

Clinical-biochemical-radiological characteristics of patients included in the final data analysis.

	**N=48**
**Age, years**	64.5 (60-71)
**BMI**	27.7 (24.5-31.1)
**Gender, M/F, n (%)**	44/4 (91.7/8.3)
**Comorbidities, n (%)**	
Smoke	9 (18.8)
Cardiovascular disease	30 (62.5)
Respiratory disease	3 (6.3)
Immunosuppressive therapy	2 (4.2)
HIV	1 (2.1)
Diabetes	5 (10.4)
Cancer	6 (12.5)
**Symptoms onset-to-hospital admission interval, days**	7 (4-10)
**Symptoms onset-to-mechanical ventilation, days**	12 (8.7-17.5)
**Chest-X-ray score**[a]	15 (13-16)
**CPAP treatment, n**	39 (81.2)
**CPAP days**	2 (1-5)
**Patients treated with MV, n**	43 (89.6)
**MV days**	15 (7-19)
**PPV days**	17.5 (9-23.7)
**Respiratory Rate, bpm**	20 (18-24)
**FiO_2**	0.8 (0.7-0.8)
**PEEP, cmH_2O**	14 (12-17.5)
**$mPEEP_{7D}$, cmH_2O**	14 (11.4-16.6)
**$mPEEP_{TOT}$, cmH_2O**	13.6 (10.5-16)
**$cPEEP_{PPV}$, $cmH_2O \cdot days$**	193.4 (123.4-279.5)
**Ppeak, cmH_2O**	33 (30-35)
**Tidal Volume, ml**	550 (500-600)
**GCS**	7 (3-15)
**PaO_2/FiO_2 ratio**	116.5 (93.7-142.5)
**ABG**	
pH	7.33 (7.27-7.4)
pCO_2, mmHg	46.5 (40-54.5)
pO_2, mmHg	86.5 (70.5-101.2)
Sat, %	98 (94.7-99)
**Prone position, n**	
yes	10 (20.8)
missing	6 (12.5)
**Mean arterial pressure, mmHg**	75 (70-80)
**Catecholamines**	
Epinephrine, n (%)	1 (0.2)
Epinephrine, mean $\mu g \cdot Kg \cdot min^{-1}$	0.04
Norepinephrine, n (%)	31 (64.6)
Norepinephrine, mean $\mu g \cdot Kg \cdot min^{-1}$	0.03 (0.09-0.14)
Dobutamine, n (%)	0
**Urinary output at day 1, ml**	930 (393-1480)
**Fluid balance at day 1, ml**	1025 (177-1817)
**WBC, $cells \cdot mm^3$**	8545 (6467-12235)
**AKI, n (%)**	

None	11 (22.9)
KDIGO class I	11 (22.9)
KDIGO class II	4 (8.3)
KDIGO class III	22 (45.8)
**CRRT, n (%)**	11 (22.9)
**Neutrophils, cells·mm^3**	8340 (5060-11000)
**Lymphocytes, cells·mm^3**	536 (413-815)
**Neutrophils-to-lymphocytes ratio**	16.3 (7.7-23.1)
**Hemoglobin, g·dl^{-1}**	12.7 (11.5-13.8)
**Hematocrit, %**	37.5 (35-41)
**Platelets, 10^3 cells·mm^3**	217.5 (174-331)
**C-reactive protein, mg·l^{-1}**	220 (126-322)
**Procalcitonin, µg·l^{-1}**	1.25 (0.27-2.6)
**S-albumin, g·dl^{-1}**	2.3 (2-2.7)
**Worst S-albumin, g·dl^{-1}**	1.7 (1.6-2)
**D-dimer, ng·ml^{-1}**	3766 (1358-13396)
**Worst D-dimer, ng·ml^{-1}**	13390 (3758-29524)
**Fibrinogen, mg·ml^{-1}**	700 (695-700)
**IL-6, ng·l^{-1}**	254 (160-1047)
**Ferritin, µg·l^{-1}**	2560 (1282-3494)
**S-creatinine, mg·dl^{-1}**	0.95 (0.78-1.41)
**S-urea, mg·dl^{-1}**	53.5 (33.7-83.7)
**Bilirubin, mg·dl^{-1}**	1.2 (1.2-1.35)
**AST, U·l^{-1}**	60.5 (42-100.7)
**ALT, U·l^{-1}**	46 (34-72.7)
**LDH, U·l^{-1}**	543 (437.5-703.7)
**Cholesterol$_{TOT}$, mg·dl^{-1}**	120 (98-144)
**HDL, mg·dl^{-1}**	19 (13-25)
**SOFA, score**	10 (8-11.75)
SOFA neurologic	3 (0-4)
SOFA respiratory	3 (3-4)
SOFA cardiovascular	3 (0-4)
SOFA kidney	0 (0-1)
SOFA liver	0 (0-0.75)
SOFA coagulation	0 (0-0.75)
**Pharmacological treatment, n (%)**	
Tocilizumab	12 (25)
Remdesivir	11 (22.9)
Hydroxychloroquine	31 (64.6)
Lopinavir/ritonavir	26 (54.2)
Steroids	14 (29.2)
**Sepsis/septic shock, n (%)**	28 (58.3)
**Microbiological isolates, n (%)**	
Enterococcus Faecium	12 (25)
Enterococcus Faecalis	12 (25)
Klebsiella Pneumoniae	5 (10.4)
Pseudomonas Aeruginosa	3 (6.3)
Staphylococcus coag. negative	5 (10.4)
Streptococcus spp.	1 (2.1)
Candida spp.	0
Serratia Marcescens	0
Enterobacter aerogenes	3 (6.3)

Staphylococcus Aureus	2 (4.2)
**LOS-ICU, days**	15 (6.25-19.75)
**LOS-Hospital, days**	17.5 (11-25)

Data are shown as median (IQR) or n (%) were indicated.

[a] Chest-X-ray score according to Brixia classification. BMI, body mass index; CPAP, continuous positive airway pressure; MV, mechanical ventilation; PPV, positive pressure ventilation; PEEP, positive end expiratory pressure; $mPEEP_{7D}$, mean PEEP applied in the first seven days since admission; $mPEEP_{TOT}$, mean PEEP applied with positive pressure ventilation during hospitalization; $cPEEP_{PPV}$, cumulative PEEP during positive pressure ventilation; Ppeak, peak airway pressure measured at admission in mechanically ventilated patients; GCS, Glasgow Coma Score; ABG, arterial blood gas analysis; WBC, white blood cells; AKI, acute kidney injury; CRRT, continuous renal replacement therapy; IL-6, plasma interleukin 6; AST, aspartate-amino transferase; ALT, alanine-amino transferase; LDH, lactic dehydrogenase; HDL, high density lipoprotein; SOFA, Sequential Organ Failure Assessment; LOS, length of stay; ICU, Intensive Care Unit.

e-Table 2

Histopathologic findings in the whole cohort (n=75) and in subgroups.

	Whole cohort (n=75)	Clinical data available (n=48)	No clinical data (n=27)	p-value
**Histopathologic patterns**				
Exudative DAD	3 (1-5)	4 (1-5.75)	2 (0-5)	0.278
Early proliferative DAD	8 (2-10)	10 (7.25-10)	0 (0-5)	<0.0001
AFOP	0 (0-1)	0 (0-1)	0 (0-0)	0.006
Interstitial pneumonia	5 (3-5)	5 (4-5.75)	4 (1-5)	0.018
Late proliferative DAD	0 (0-4)	2 (0-5)	0 (0-0)	<0.0001
Bronchopneumonia	2 (0-5)	3 (0-6)	0 (0-4)	0.03
Thrombi	3 (0-5)	3 (2-5.75)	0 (0-4)	0.02
Megakaryocytes	6 (5-10)	6 (5-10)	5 (4-7)	0.031
Normal lung areas	0 (0-5)	0 (0-0)	7 (1-10)	<0.0001
**Other findings, n (%)**				
Aspergillus	5 (6.7)	3 (6.3)	2 (7.4)	0.99
Giant cells	7 (9.3)	7 (14.6)	0	0.045
Hemorrhage	13 (17.3)	9 (18.8)	4 (14.8)	0.76
Fibrosis	5 (6.7)	3 (6.3)	2 (7.4)	0.99
Pleuritis	5 (6.7)	5 (10.4)	0	0.15
Infarct	13 (17.3)	11 (22.9)	2 (7.4)	0.11
Lympho-plasma cells Alveolar infiltrates	3 (4)	3 (6.3)	0	0.55
Edema	8 (10.7)	2 (4.2)	6 (22.2)	0.022

Data are shown as median (IQR) or n (%) where indicated. P-values were calculated by Mann-Withney test.

e-Figure 1

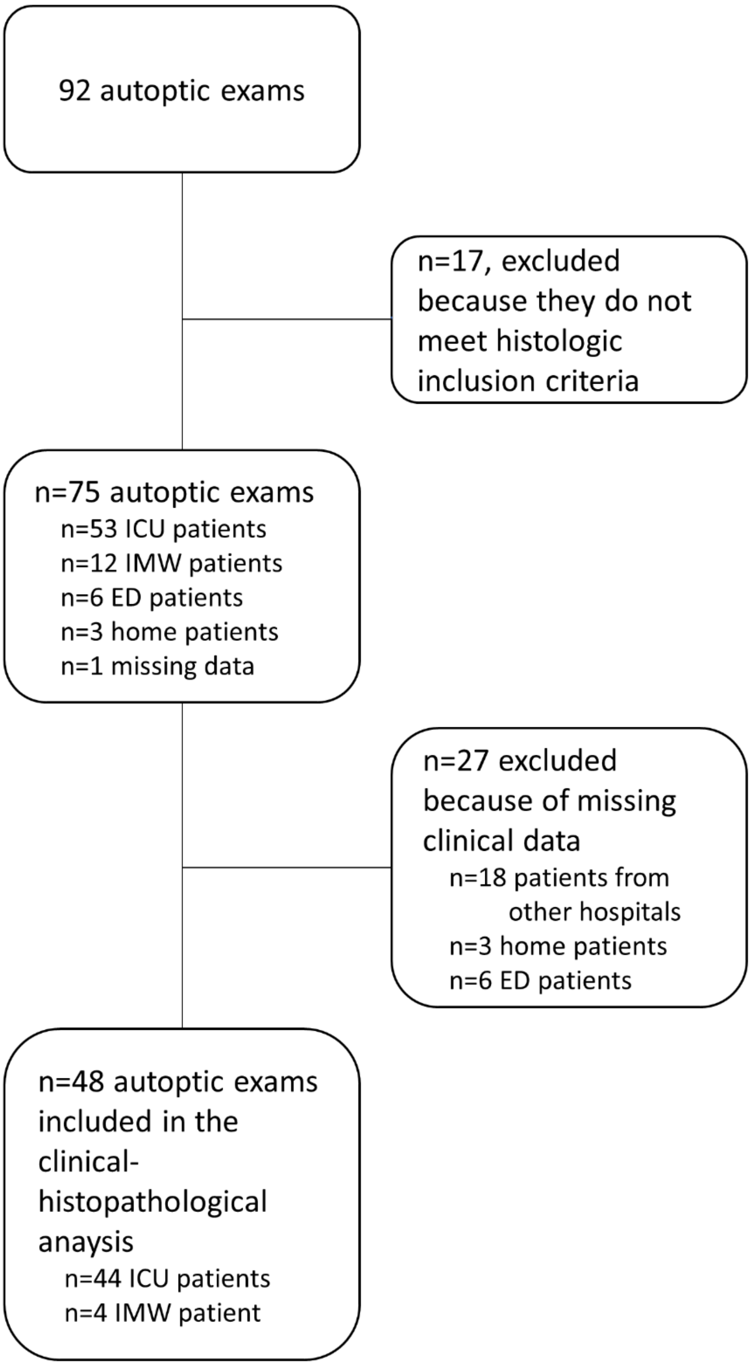

Flow chart of the patient selection. 92 autopsies were performed during the predefined study period. 75 had full histopathologic examination according to the inclusion criteria, 48 had also complete clinical records. *ICU*, intensive care unit; *IMW*, internal medicine ward; *ED*, emergency department.

e-Figure 2

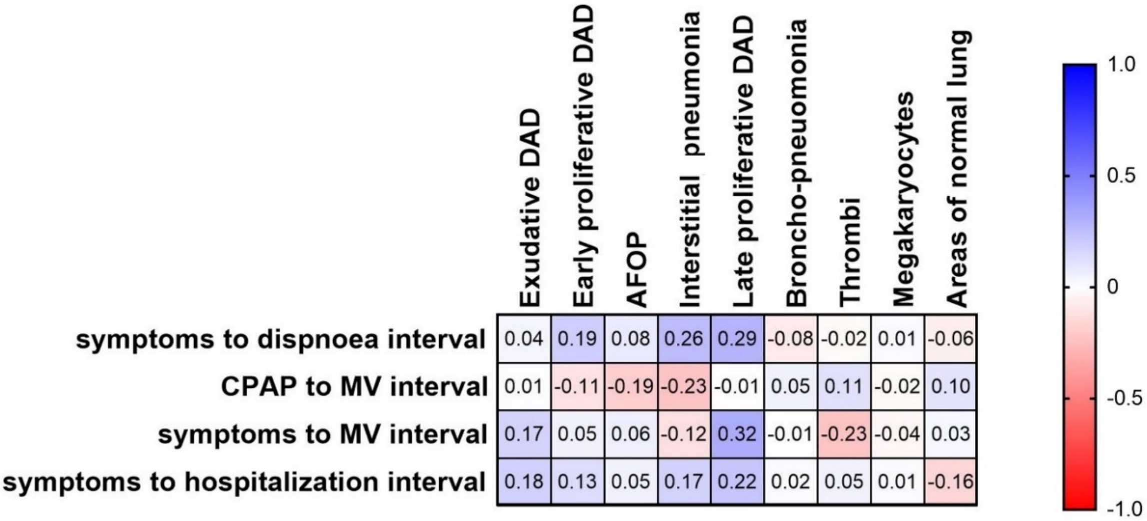

Correlation matrix (Spearman's r) *between* intervals from i) symptoms to dispnoea, ii) CPAP to MV, iii) symptoms to MV, iv) symptoms to hospitalization *and* the histopathologic patterns. Correlation coefficients are shown into the cells and none of them was significant.

e-Figure 3

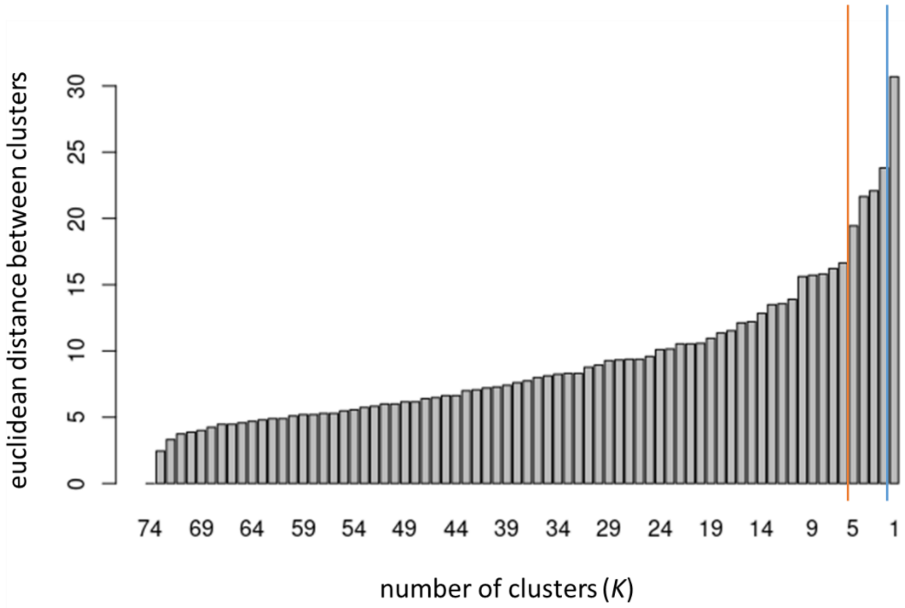

Choice of the number of clusters (*K*). According to the "elbow" method, the difference of heights plotted in the figure shows that the largest jumps of heights was between 1 and 2 clusters (blue line) and between 5 and 6 clusters (orange line). Two-cluster based analysis was able to discriminate almost all patients with clinical information from those without it. In order to characterize the histopathologic cluster, the second "elbow" was more representative.

e-Figure 4

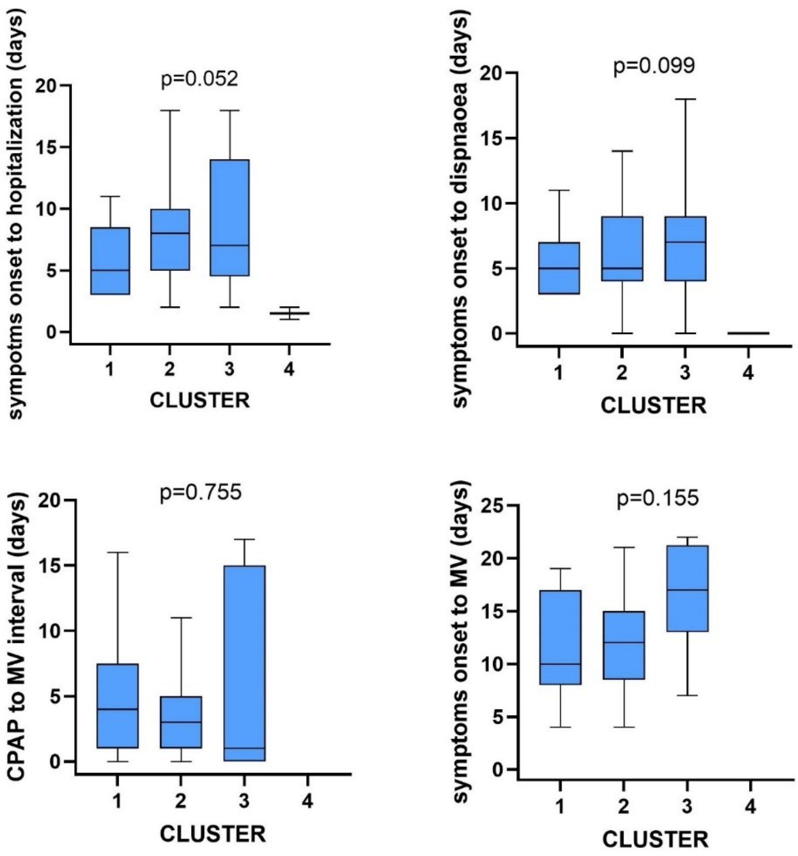

Distribution of intervals from symptoms onset to hospital admission, from symptoms to dispnoea onset, from symptoms onset to MV start, and from CPAP to MV start among clusters. P-values were calculated with Kruskal-Wallis test (see Methods in the text).

e-Figure 5

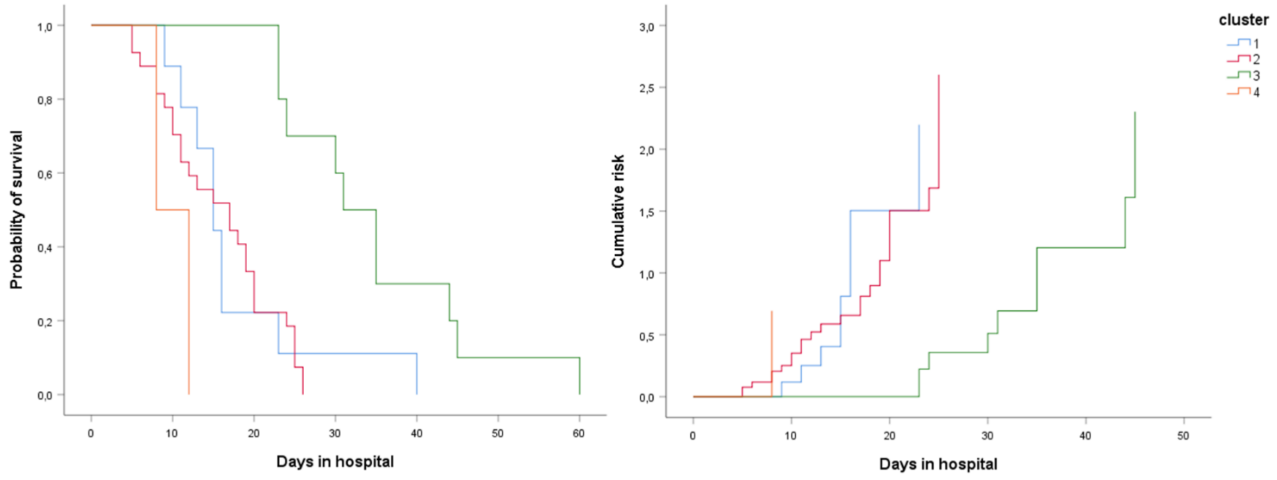

Detailed Kaplan Meier curves of clusters of patients with full clinical records as shown in Figure 6 in the text. p<0.0001 (Log Rank Mantel–Cox test).

e-Figure 6

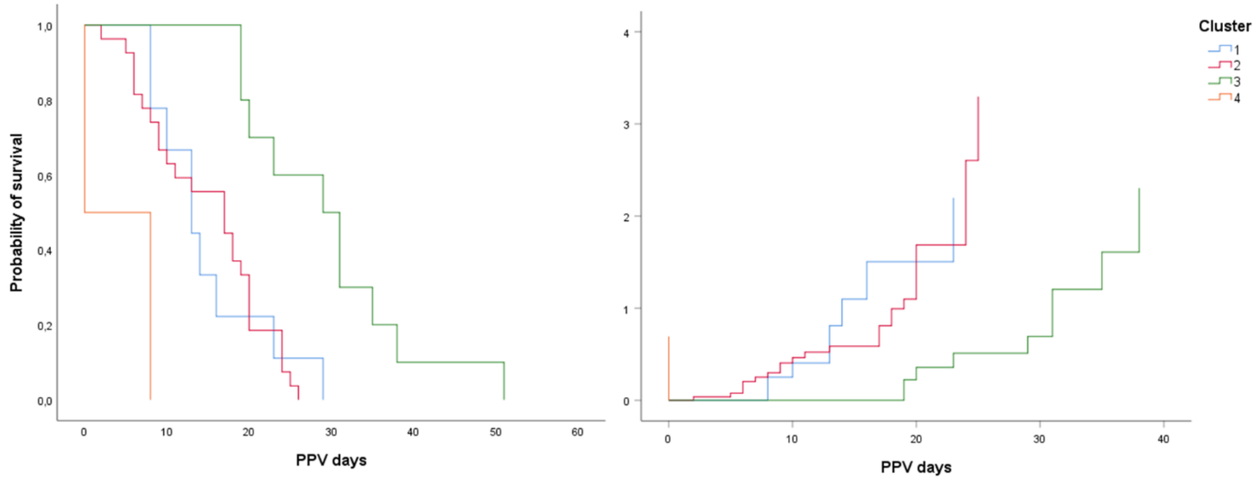

Kaplan Meier curves of clusters of patients with full clinical records who underwent positive pressure ventilation (PPV) P<0.0001 (Log Rank Mantel–Cox test).

e-Figure 7

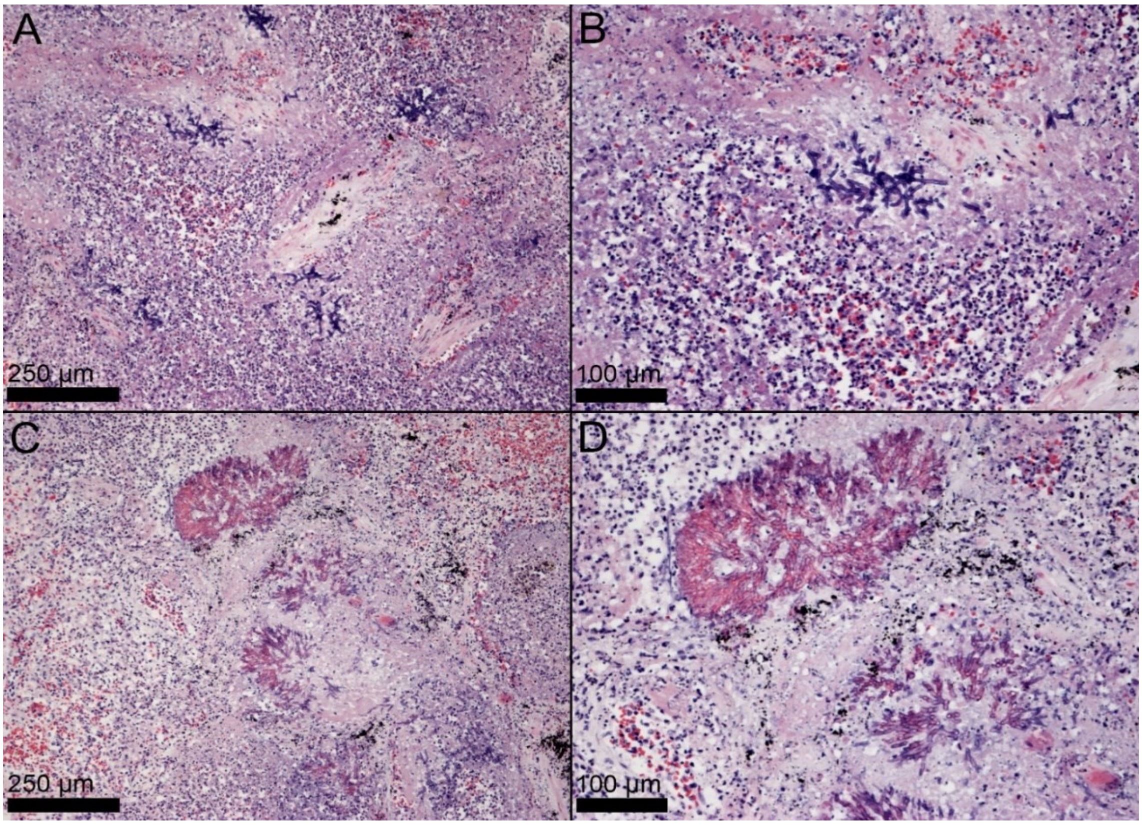

Histopathology of a case with aspergillosis. Areas of acute inflammatory infiltrate, cellular debris, and scattered basophilic hyphae (A, 10x, H&E; B, 20x, H&E) admixed with other areas with florid eosinophilic hyphae (C, A, 10x, H&E; D, 20x, H&E) in case n. 8.
